# Underwater Superaerophobic and Superaerophilic Nanoneedles‐Structured Meshes for Water/Bubbles Separation: Removing or Collecting Gas Bubbles in Water

**DOI:** 10.1002/gch2.201700133

**Published:** 2018-04-25

**Authors:** Jiale Yong, Feng Chen, Wentao Li, Jinglan Huo, Yao Fang, Qing Yang, Hao Bian, Xun Hou

**Affiliations:** ^1^ State Key Laboratory for Manufacturing System Engineering and Key Laboratory of Photonics Technology for Information of Shaanxi Province School of Electronics and Information Engineering Xi'an Jiaotong University Xi'an 710049 P. R. China; ^2^ School of Mechanical Engineering Xi'an Jiaotong University Xi'an 710049 P. R. China

**Keywords:** superhydrophilicity, superhydrophobicity, underwater superaerophilicity, underwater superaerophobicity, water/bubbles separation

## Abstract

Water/bubbles separation has great practical significance, which can avoid the harm caused by underwater bubbles or cleverly collect useful gas bubbles in water. Here, a Cu(OH)_2_‐nanoneedles‐structured rough copper mesh is fabricated by a one‐step chemical reaction. The original rough mesh shows superhydrophilicity in air and superaerophobicity in water. The underwater superaerophobic mesh has great anti/removing‐bubbles ability in water. In contrast, the rough mesh switches to superhydrophobicity in air and superaerophilicity in water after further being modified with fluoroalkylsilane. The underwater superaerophilic mesh can absorb bubbles and allow bubbles to pass through the mesh. Based on the superhydrophilic/superaerophobic mesh and the superhydrophobic/superaerophilic mesh, a strategy to remove gas bubbles from the water pipe is proposed, and an in‐water bubbles‐collection device is also designed. It is believed that these two kinds of mesh will have more applications in controlling the behavior of underwater bubbles.

## 1 Introduction

Gas can appear in water in the form of bubbles. Separating liquid and small gas bubbles has great applicable value, which can avoid the harm caused by those bubbles or cleverly collect useful gas bubbles in a liquid medium.^1^, ^2^, ^3^, ^4^, ^5^, ^6^ If a solid surface has great bubble‐repellent ability, it will be able to solve some bubbles‐induced problems. For example, bubbles that are generated in a microfluidic system can increase the fluid frictional loss and even be possible to block the microchannels. In a gas‐generating electrochemical reaction, if a lot of generated bubbles adhere to the electrode, the reaction efficiency will be greatly reduced.^7^, ^8^, ^9^, ^10^ Excess bubbles entering into human blood vessels during vein injection will endanger the lives of patients because of embolism. Bubbles' adhesion usually results in a blurred vision of the underwater camera and diving goggle as well as the in‐air water container (e.g., fish tank). By contrast, if a material has bubbles‐absorption capacity, it can be used to collect underwater useful gas bubbles.^11^, ^12^ For example, methane gas in particular places is constantly seeping out of the earth's interior and continues to rise to the surface of water in the form of bubbles, releasing methane into the atmosphere.^13^ The capture and utilization of such self‐escaping methane bubbles can contribute to meeting the great demand for energy sources.^11^ In the process of photocatalytic decomposition of water to hydrogen, a critical thing is to collect generated hydrogen bubbles in time.^12^ In various industrial wastewaters, there are often huge amounts of toxic sulfide gas and ammonia, so pre‐collecting these gas bubbles is helpful to decrease environment pollution caused by wastewater discharge.

The ability of removing or collecting bubbles for a solid material is closely related to the behavior of bubbles on such material surface in a water medium, especially for the two extreme cases: underwater superaerophobicity and underwater superaerophilicity. Similar to the definition of superhydrophobicity and superhydrophilicity, underwater superaerophobic surface is generally defined as exhibiting contact angle (CA) to a gas bubble larger than 150° in water, while underwater superaerophilic surface is generally defined as exhibiting CA to a gas bubble less than 10°.^14^, ^15^, ^16^, ^17^, ^18^, ^19^, ^20^, ^21^, ^22^, ^23^, ^24^, ^25^, ^26^ The research on the underwater superaerophobicity and superaerophilicity is only beginning and is now still at the “toddler stage” of development.^2^, ^3^ To the best of our knowledge, simple achievement of water/bubbles separation by using underwater superaerophobic/superaerophilic porous materials has rarely been reported until now.

In this paper, we report a new approach for the fabrication of underwater superaerophobic and superaerophilic meshes, respectively. A micro/nanoscale hierarchical rough copper mesh that was covered by Cu(OH)_2_ nanoneedles was obtained by a one‐step chemical reaction. The original rough mesh showed superhydrophilicity in air and superaerophobicity in water. After further being modified with fluoroalkylsilane, the rough mesh switched to superhydrophobicity in air and superaerophilicity in water. The underwater superaerophobic mesh has great antibubble ability in water; therefore, it can prevent bubbles from passing through the mesh and remove gas bubbles from water. In contrast, the underwater superaerophilic mesh can absorb bubbles and allow the bubbles to pass through the mesh. By using the as‐prepared underwater superaerophobic and superaerophilic meshes as the core component, we proposed a novel strategy to remove gas bubbles from the water pipe (or microchannel), and we also designed a device that can collect gas bubbles in water.

## 2 Results and Discussion

### 2.1 Nanoneedles‐Structured Copper Meshes

**Figure 1:**
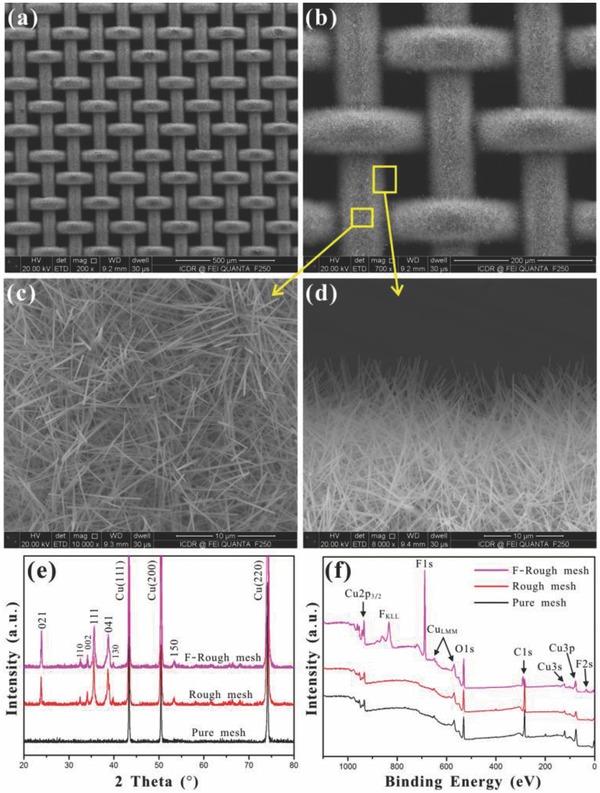
Microstructure and chemical composition of the nanoneedles‐structured rough copper mesh. a–d) SEM images of the rough mesh after reaction. e) XRD patterns and f) XPS patterns of the pure copper mesh, rough mesh, and F‐rough mesh, respectively.

Copper mesh was used as the substrate material because of its inherent porous microstructure and good corrosion resistance which is important for underwater applications. The pure copper mesh (200 mesh size) has an inherent microscale rough structure with an average pore diameter of ≈76 µm (Figure S1, Supporting Information). Its surface is smooth and clean. A layer of nanoneedles was simply formed on the surface of copper wire by immersing a copper mesh into the solution of NaOH and (NH_4_)_2_S_2_O_8_. **Figure** **1**a–d shows the scanning electron microscopy (SEM) images of the copper mesh after reaction for 5 min. The whole mesh is uniformly and completely covered by the nanoneedles. Such nanoneedles are about 10–15 µm in length and 150–260 nm in diameter, and grow vertically on the copper wires and interlace with each other (Figure 1c,d, Figure S2, Supporting Information). X‐ray diffraction (XRD) analysis was performed to further study the chemical changes and crystal structure of the as‐prepared nanoneedles. Figure 1e shows the XRD patterns of the pure cooper mesh and the rough mesh. Before reaction, only the Cu phase appears. New peaks show up after the growth of the nanoneedles on the copper mesh substrate. Those peaks are in agreement with the peak position of the orthorhombic Cu(OH)_2_, indicating that the as‐synthesized nanoneedles are composed of Cu(OH)_2_ crystals.^27^, ^28^ Cu(OH)_2_ is a typical layered material. Its orthorhombic crystal phase has proven to be suitable for the assembly of nanoribbons and nanowires.^29^, ^30^, ^31^, ^32^ It is well known that Cu^2+^ prefers square planar coordination by OHˉ, leading to an extended chain. The chains tend to be connected through the coordination of OH^−^ to dz^2^ orbital of Cu^2+^, resulting in a 2D structure.^29^ In addition, the 2D layers are stacked through the relatively weak hydrogen bond interactions, and form a 3D crystal. Once the pure copper mesh was lightly placed into the reaction solution, the oxidation reaction started immediately and the Cu(OH)_2_ nanoneedles appeared. Many experiments have also demonstrated that the surface microstructures (i.e., the length and the density) of the generated Cu(OH)_2_ nanoneedles are greatly influenced by the reaction time.^27^, ^28^ In our experiment, when the reaction time increased to about 5 min, the nanoneedles can completely wrap on the copper mesh. For convenience, the nanoneedles‐structured copper mesh is defined as “rough mesh,” When such rough mesh was further immersing in the solution of fluoroalkylsilane, we would obtain a fluorinated rough mesh, defined as the “F‐rough mesh.” The fluorination process did not change the crystal structure of the rough mesh (Figure 1e). X‐ray photoelectron spectroscopy (XPS) analysis was carried out to determine the surface composition of the pure copper mesh, rough mesh, and F‐rough mesh, respectively. As shown in Figure 1f, only copper, oxygen, and carbon were detected on both the pure copper mesh and the rough mesh. The peaks at 932.3 and 953.2 eV are ascribed to Cu 2p3/2 and Cu 2p1/2 of Cu^2+^, respectively. However, an obvious fluorine signal was also detected on the F‐rough mesh except the above‐mentioned three elements. The peak at about 688.2 eV is attributed to F 1s in the fluoroalkylsilane, confirming that a stable fluoroalkylsilane monolayer was successfully modified onto the surface of rough mesh.

### 2.2 Underwater Superaerophobicity and Superaerophilicity

**Figure 2:**
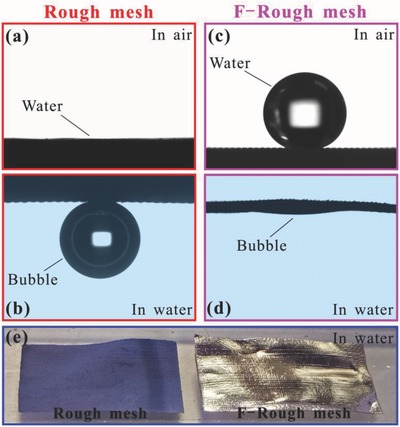
Static wettability of a rough mesh and an F‐rough mesh, respectively. a) Water droplet on a rough mesh in air. b) Bubble on a rough mesh in water. c) Water droplet on an F‐rough mesh in air. d) Bubble on an F‐rough mesh in water. e) Photograph of a rough mesh and an F‐rough mesh immersing in water.

**Figure 3:**
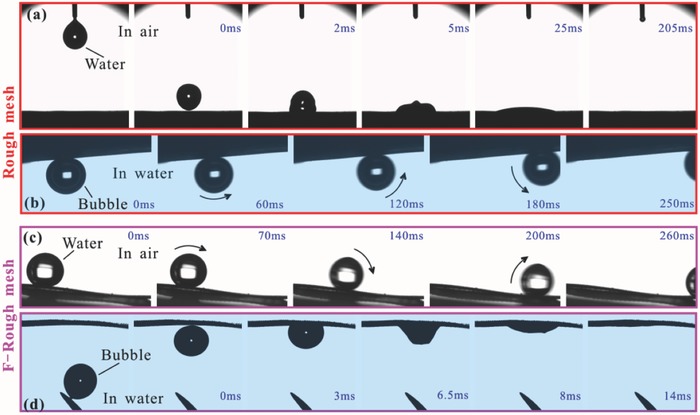
Dynamic wettability of a rough mesh and an F‐rough mesh, respectively. a) Water droplet spreading out on a rough mesh in air. b) Bubble rolling on a rough mesh in water. c) Water droplet rolling on an F‐rough mesh in air. d) Bubble being absorbed by an F‐rough mesh in water.

When a water droplet was dripped onto the rough mesh, the water droplet would spread out quickly and be absorbed by the mesh within 205 ms, resulting in a water CA of ≈0° (**Figure** **2**a, **Figure** **3**a, Movie S1, Supporting Information). Therefore, the rough mesh is superhydrophilic in air. Since Cu and Cu(OH)_2_ are intrinsically hydrophilic, the rough microstructure of the mesh and the Cu(OH)_2_ nanoneedles can amplify the hydrophilicity to superhydrophilicity.^4^, ^5^, ^6^ The left part of Figure 2e shows the photo of a rough mesh underwater. The rough mesh is fully wetted by water and shows a blue color, agreeing well with the inherent color of Cu(OH)_2_. The mesh was still wet after taking out of water (Movie S2, Supporting Information). In a water medium, when a gas bubble was released onto the rough mesh, the bubble could maintain an approximately spherical shape with the gas CA of 161.5° ± 2.5°, revealing the underwater superaerophobicity of the rough mesh, as shown in Figure 2b. The rough mesh also exhibited ultralow adhesion to the bubble in water, because the bubble could roll off as soon as the rough mesh was tilted 5.5° (Figure 3b and Movie S3, Supporting Information). This result indicates that the rough mesh has antibubble ability in water.

Contrary to the superhydrophilicity of the original rough mesh, the F‐rough mesh showed superhydrophobicity with a water CA of 153° ± 3° (Figure 2c) and water sliding angle of 6.5° (Figure 3c, Movie S4, Supporting Information) in air environment. A silver mirror‐like reflectance appeared after immersing such mesh in water, as shown in the right part of Figure 2e. When the mesh was taken out of the water, it still remained dry (Movie S2, Supporting Information). This result reveals that a water droplet on the F‐rough mesh is at the Cassie–Baxter wetting state because the mirror‐like interface is resulted from a trapped air layer between water and the mesh surface,^33^, ^34^, ^35^ as observed on *Salvinia* plants.^36^ In water, the F‐rough surface had different wettability to a gas bubble compared to the rough mesh. It could absorb rather than repel bubbles. When a bubble was released below the F‐rough mesh, it would rise up in water. The bubble would spread out quickly once it contacted with the mesh, resulting in a very small gas CA near 6.5°, revealing the underwater superaerophilicity of the F‐rough mesh (Figures 2d and Figure 3d, and Movie S5, Supporting Information).

**Figure 4:**
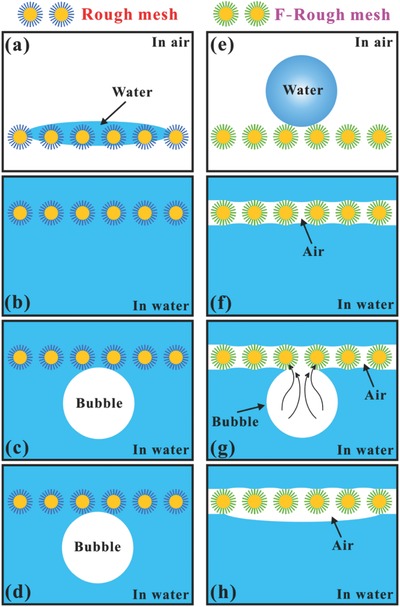
Formation mechanisms of the underwater superaerophobicity of a–d) the rough mesh and the underwater superaerophilicity of e–h) the F‐rough mesh.

The formation mechanisms of the underwater superaerophobicity of the rough mesh and the underwater superaerophilicity of the F‐rough mesh are depicted in **Figure** **4**. The underwater superaerophobicity and superaerophilicity are closely related to the in‐air water wettability of those meshes. The rough mesh is superhydrophilic in air; a water droplet that is dripped onto the mesh can wet the mesh and is at the Wenzel state (Figure 4a).^21^, ^37^, ^38^, ^39^, ^40^, ^41^ Both the micropores between the copper wires and the space between the nanoneedles are fully wetted by water after such rough mesh being immersed in water (Figure 4b). The water occupying the interspaces between the microstructures may seem to be trapped by the rough mesh. As shown in Figure 4c, when a gas bubble is placed on the rough mesh, the trapped water layer will prevent the bubble from effectively contacting the rough mesh, because gas and water generally repel each other. The trapped water layer is tightly attracted by the rough mesh and is hard to be replaced by the foreign gas bubble. In fact, the underwater bubble sits on the mesh surface and can only contact with the peaks of the microstructures.^42^, ^43^ The bubble is surrounded by water, so it still retains a sphere shape on the rough mesh, yielding underwater superaerophobicity of the rough mesh (Figure 4c). The shape of the spherical bubble does not change as time goes on (Figure 4d). In such water/gas/solid three‐phase system, the bubble's behavior can be regarded as an underwater version of Cassie–Baxter state.^2^, ^3^ The underwater superaerophobicity of the rough mesh has a similar formation mechanism with the underwater superoleophobicity of in‐air superhydrophilic materials.^20^, ^21^, ^44^, ^45^ After the modification of the rough mesh with low‐surface‐energy fluoroalkylsilane, its wettability switches from superhydrophilicity to superhydrophobicity in air. Meanwhile, the underwater behavior of a bubble on the mesh also changes greatly. The micro/nanoscale hierarchical rough structures and the low‐surface‐energy chemical composition endow the F‐rough mesh with superhydrophobicity and ultralow water adhesion. A water droplet on such mesh is at the Cassie wetting state (Figure 4e).^21^, ^44^, ^46^, ^47^, ^48^, ^49^, ^50^ The droplet only touches the peaks of the rough microstructures, resulting in an air cushion being trapped between the mesh surface and the water droplet. When the F‐rough mesh is dipped into water, the air cushion beneath the water droplet will become a trapped air layer surrounding the mesh, as shown in Figure 4f.^51^, ^52^ If a bubble touches the underwater F‐rough mesh, the gas in the bubble will merge with the air in the trapped air layer (Figure 4g). The bubble spreads out along the trapped air layer under pressure. It looks to be completely absorbed by the mesh; thereby, the F‐rough mesh shows underwater superaerophilicity (Figure 4h). In fact, the underwater superaerophobicity of the rough mesh is ascribed to its in‐air superhydrophilicity, while the underwater superaerophilicity of the F‐rough mesh is ascribed to its in‐air superhydrophobicity. The underwater superaerophobicity endows the rough mesh with antibubble ability, while the underwater superaerophilicity endows the F‐rough mesh with gas‐absorption capacity in water.

### 2.3 Intercepting Bubbles or Allowing Bubbles to Pass Through

**Figure 5:**
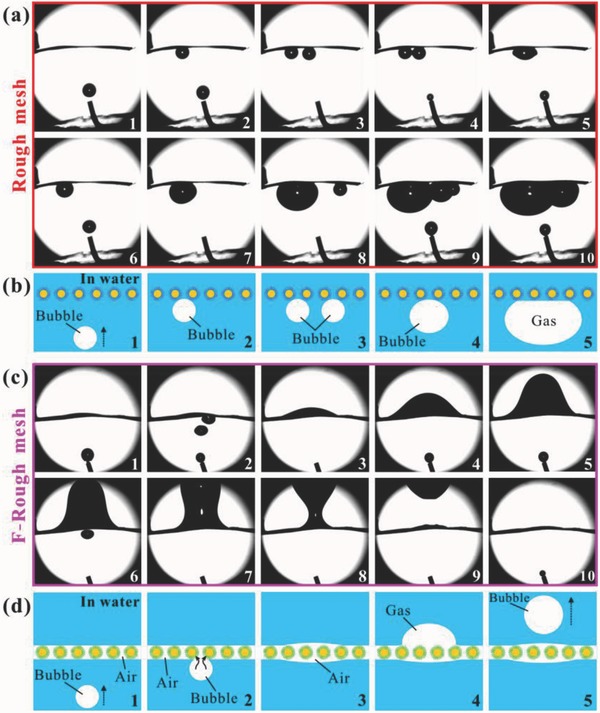
Bubbles selectively passing through the as‐prepared meshes. a) Bubbles being intercepted by the underwater rough mesh. b) Schematic illustration of the process in (a). c) Bubbles passing through the underwater F‐rough mesh. d) Schematic illustration of the process in (c).

The condition of a large amount of bubbles rising to the as‐prepared meshes in water was also investigated. As shown in **Figure** **5**a and Movie S6 (Supporting Information), when the first bubble reached to the rough mesh, it was intercepted by the rough mesh and stopped to rising up (Steps 1–2). The second bubble was also unable to pass through the underwater rough mesh (Step 3). Once the second bubble contacted with the first one, they would merge into a bigger one (Steps 4–6). With more and more bubbles flowing to the below side of the rough mesh, all of them were stopped by the mesh; that is, there was no one passing through the mesh. The merged bubbles became bigger and bigger (Steps 7–10). Therefore, the rough mesh has the ability to prevent underwater gas bubbles from passing through. In fact, this result also demonstrated that the rough mesh could remove the gas bubbles in water. The bubbles‐interception function of the rough mesh is the direct result of its underwater gas repellency caused by the underwater superaerophobicity. As shown in Figure 5b, no matter how many bubbles rise up to the bottom side of the rough mesh, the underwater superaerophobicity allows the mesh to repel all of the bubbles. The bubbles can only merge into a new one and continue to grow.

Contrary to the underwater rough mesh, when the first few bubbles contacted with the underwater F‐rough mesh, they would be instantly absorbed by the mesh (Steps 1–2), agreeing well with the underwater superaerophilicity of the F‐rough mesh, as shown in Figure 5c and Movie S7 (Supporting Information). Then, the trapped gas layer in the microstructures bulged on the top side of the mesh (Step 3). As more and more bubbles reached to the mesh, the gas bulge was continuously growing, with its shape changing from “crescent” to “bell” and then to “wine goblet” (Steps 4–8). When the gas bulge was large enough after accumulation, the buoyancy force acting on the gas bulge could break the tension force between the gas bulge and the gas trapped in the mesh. As a result, the gas bulge got rid of the bondage of the mesh and rose up again (Steps 9–10), one after another. All of the bubbles could pass through the mesh in water. It is the underwater superaerophilicity that allows the bubbles to be absorbed and further pass through the F‐rough mesh. Figure 5d is the schematic illustration of the process of bubbles passing through the underwater F‐rough mesh. The F‐rough mesh is superhydrophobic and underwater superaerophilic. When the mesh is immersed in water, air can be trapped in both the surface microstructure and the through microholes. A trapped air layer is formed which connects the bottom and top surfaces of the underwater mesh.^33^, ^34^, ^35^ When a bubble contacted the mesh, the air in the bubble would quickly merge with the trapped air layer and enter into the inner space of the mesh, resulting in that the bubble is absorbed by the F‐rough mesh. As more and more bubbles were absorbed by the mesh and entered into the trapped gas layer, the pressure of the trapped gas layer was increasing. After enough accumulation, the pressure in the trapped air layer is big enough to allow the trapped air to lift the water that pressed on the top side of the F‐rough mesh and come out of the mesh, resulting in a big gas bulge. With the volume of the air bugle increasing, the gas bulge could finally leave the top mesh surface and rose up in the form of a big bubble by the driving force of buoyancy. Such underwater superaerophilic mesh can absorb bubbles and also can allow the gas bubbles to pass through. It has important potential applications in underwater gas collection and underwater filtrate.

### 2.4 Removing Bubbles in Water Pipe (or Microchannel)

**Figure 6:**
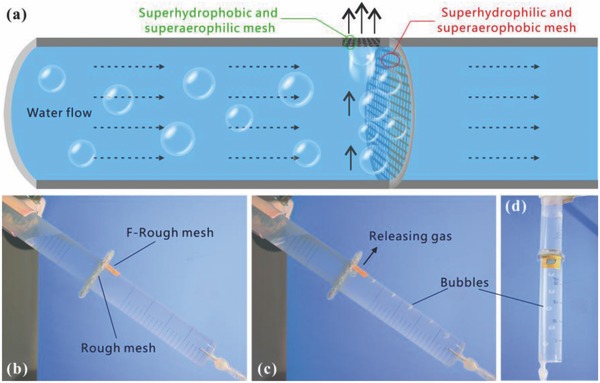
Removing bubbles in a water pipe. a) Schematic diagram of the designed device and the working principle for removing bubbles. b) A man‐made bubbles‐removing water pipe filled with water. Images of introducing continual bubbles in the lower end of the designed device: c) side view and d) top view. To clearly observe the bubbles, a blue plastic plate was used as background.

Bubbles in water pipes or microfluidic system usually result in big fluid frictional loss and local resistance, and can even block the microchannels. At present, the as‐prepared metal meshes with extreme wettability provide a promising way to solve this problem. **Figure** **6**a shows the schematic diagram of removing bubbles in a water pipe. A superhydrophilic and underwater superaerophobic rough mesh is fixed inside the pipe, which is perpendicular to the pipeline. The superhydrophilicity allows water to wet and flow through the mesh. However, bubbles in water are intercepted by the rough mesh due to the underwater superaerophobicity. As a result, bubbles gather together before this mesh. There is a through hole on the top of the pipe, and a superhydrophobic and underwater superaerophilic F‐rough mesh is covered over this hole. The superhydrophobicity can prevent the water in the pipe from flowing out, while the underwater superaerophilicity allows the gathered gas to pass through the F‐rough mesh and discharge from the pipe. In this way, the bubbles in water flow can be removed by the combined effect of these two different kinds of meshes. Figure 6b shows a man‐made pipeline which was designed based on our proposed structure. An as‐prepared rough mesh was sandwiched between two plastic tubes. The top of the tube before the mesh was drilled to form a through hole near the rough mesh. The hole was further covered by an F‐rough mesh. When water was poured into one end of the tube, the water could cross the rough mesh (Figure S3 and Movie S8, Supporting Information). Finally, both tubes were full of water. Although a hole was on the wall of the tube, the water in the pipe did not burst out (Figure 6b). As shown in Figure 6c,d and Movie S8 (Supporting Information), if bubbles were introduced into the lower end of the designed device, the bubbles would rise up along the pipeline until reaching the rough mesh. The bubbles in water flow were intercepted by this mesh, and were released outside of the pipeline by passing through the hole and the F‐rough mesh. There were no bubbles appearing in the water of the tube at another side of the rough mesh, demonstrating that bubbles in water flow were successfully removed.

### 2.5 Collecting Bubbles in Water

**Figure 7:**
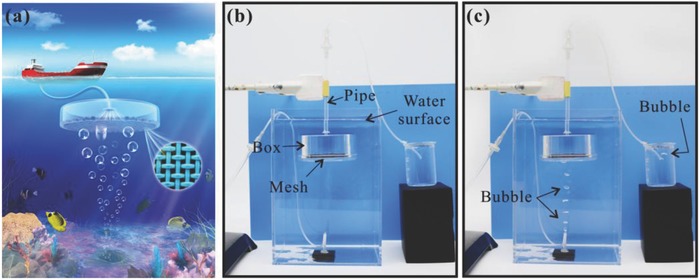
Collecting bubbles in water. a) Schematic diagram of the designed device for collecting bubbles in water. Proof‐of‐concept experiment of collecting bubbles by the designed device: b) before releasing bubbles and c) after releasing bubbles. To clearly observe the bubbles, a blue plastic plate was used as background.

Based on the special wettability of the as‐prepared F‐rough mesh that simultaneously repels water and can allow bubbles to pass through, we designed an in‐water gas bubbles‐collection device, as shown in **Figure** **7**a. The device is mainly composed of three parts: a cylindrical box without bottom, a transporting pipe, and a bottom mesh. The box forms the backbone of the device. An F‐rough mesh as the bottom mesh is covered on the bottom of the box to enclose the box. A plastic pipe extends from the inner to the outside of the closed box, which is used to transport the collected gas to users. The most critical component of this device is the F‐rough mesh bottom. The superhydrophobicity of the F‐rough mesh can prevent water from wetting the mesh and entering into the collection device, while its underwater superaerophilicity allows gas bubbles to pass through the bottom mesh and endows the device with bubble‐collecting ability. A proof‐of‐concept experiment was performed. As shown in Figure 7b, the designed device was put into a big beaker full of water; the output of the pipe was inserted into another small beaker filled with water to monitor whether the gas output or not. Once a large amount of air bubbles was continually released to the bottom of the big beaker, the bubbles would rise up and be absorbed by the bottom mesh of the device (Figure 7c, Movie S9, Supporting Information). Meanwhile, bubbles appeared in the small beaker, demonstrating that those bubbles in big beaker were successfully collected and could be transported along the pipeline. Because the bubbles‐collection process is more involved the physical property of gas bubbles but nothing to do with the gas species, therefore, such device can potentially collect various kinds of gas bubbles. For example, a large amount of methane gas is constantly self‐seeping out of the seafloor and releasing into the atmosphere as it rises to the water surface in the form of bubbles. Such self‐release makes a big waste of energy sources. This designed device can potentially be used to collect the self‐escaping methane bubbles from seafloor (Figure 7a), before the release of the bubbles into the atmosphere.

### 2.6 Influence of the Pore Diameter of the Mesh and the Submergence Depth in Water

**Figure 8:**
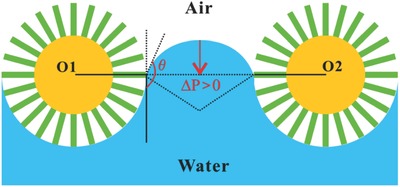
Schematic diagram of the wetting model of the F‐rough mesh in water.

The bubbles‐collection ability of the superhydrophobic and underwater superaerophilic mesh has been demonstrated. The superhydrophobicity plays a very important role in the formation of underwater superaerophilicity as well as the bubbles‐collection ability for the F‐rough mesh. The F‐rough copper mesh can allow underwater bubbles to pass through resulted from the continuous air layer trapped on the mesh in a water medium. The trapped air film underwater is greatly influenced by the pore diameter of the mesh, the submergence depth in water, the surface wettability, and the surface tension of the aqueous media. As shown in **Figure** **8**, an intrusion pressure (Δ*P*) must be exceeded before water will penetrate pores, which can be described as^53^, ^54^, ^55^, ^56^

(1) $$\Delta P=\frac{2\gamma }{R}=-\frac{l\gamma \cos\theta_{\mathrm{adv}}}{A}$$

where γ is the surface tension of water, *R* is the radius of the meniscus, *l* is the perimeter of the pores, θ_adv_ is the advancing contact angle of water on the F‐rough mesh surface, and *A* is the area of the pores. Clearly, *A* is the governing expression. This equation reveals that the intrusion pressure of the F‐rough mesh in water increases with decreasing the diameter of the micropores. A high intrusion pressure will enable the collecting device to have a large depth of submergence and high stability. Therefore, a too large submergence depth will likely lead to the loss of the superhydrophobicity and underwater superaerophilicity of the F‐rough mesh, but this problem can be solved by properly decreasing the pore diameter of the mesh. In addition, we need only collect underwater bubbles slightly below water surface because bubbles can spontaneously rise to the top of water.

## 3 Conclusions

In conclusion, a layer of Cu(OH)_2_ nanoneedles was generated on a copper mesh by simply immersing the copper mesh in the solution composed of NaOH and (NH_4_)_2_S_2_O_8_. The obtained rough mesh was superhydrophilic in air, while it became superaerophobic after immersion in water. Such rough mesh could intercept the gas bubbles due to its underwater superaerophobicity and antibubble ability, preventing bubbles from passing through the mesh. Therefore, the mesh has bubbles‐removing function in a water medium. Once the rough mesh was further modified with fluoroalkylsilane, the resultant F‐rough mesh would show superhydrophobicity in air and superaerophilicity in water. The underwater superaerophilicity endowed the mesh with gas‐absorption ability in water and allowed the bubbles to pass through the mesh. By combining an underwater superaerophobic mesh to intercept bubbles and an underwater superaerophilic mesh to discharge gas, a strategy to remove gas bubbles from the water pipe (or microchannel) was proposed. In addition, an in‐water bubbles‐collection device was designed based on the superhydrophobic and underwater superaerophilic F‐rough mesh. This device has potential application in collecting self‐escaping methane gas from seafloor, to alleviate the pressure of the growing energy demands. We believe that the designed underwater superaerophobic/superaerophilic meshes will have important applications in controlling bubbles' behavior on a solid surface in water, excluding bubbles‐induced hazards, and the clever use of underwater bubbles in some cases.

## 4 Experimental Section

*Preparation of Nanoneedles‐Structured Rough Mesh*: The nanoneedles‐structured copper mesh was prepared by a similar method as previously reported.^27^, ^28^ The copper mesh substrates (99.9%) with a mesh number of 200 were ultrasonically cleaned with acetone, ethanol, and deionized water. Then, the meshes were immersed in an aqueous solution of NaOH (2.5 m) and (NH_4_)_2_S_2_O_8_ (0.1 m) at room temperature for 5 min. The meshes were taken out and washed with deionized water, and dried under N_2_. The as‐prepared rough mesh turned to blue from the original red color.

*Preparation of the F‐Rough Mesh*: The nanoneedles structured rough meshes that were directly obtained by chemical reaction were immersed into a 0.2% fluoroalkylsilane solution (in ethanol) for 24 h in order to modify a molecular layer with low surface free energy. Immediately afterward, the meshes were stored in a vacuum oven at 100 °C for 2 h to make the fluoride layer more stable. For convenience, the nanoneedles structured copper mesh is defined as “rough mesh.” When such rough mesh was further modified in the solution of fluoroalkylsilane, a fluorinated rough mesh, defined as the “F‐Rough mesh,” was obtained.

*Preparation of the Bubbles‐Removing Water Pipe*: A resultant rough mesh was sandwiched between two plastic tubes. The end of the tube before the mesh was drilled to form a through hole near the rough mesh, and an F‐rough mesh was covered over this hole.

*Preparation of the Bubbles Collection Device*: The device is composed of three parts: a cylindrical box without bottom, a pipe, and a bottom mesh. The box forms the backbone of the device. An F‐rough mesh as the bottom mesh was covered on the bottom of the box to enclose the box. The thin pipe extended from the inner to the outside of the closed box, which was used to transport the collected gas to users.

*Characterization*: The surface morphology of the rough mesh was observed by a scanning electron microscope (Quantan 250 FEG, FEI, America). The CAs and sliding angles of water droplet (≈6 µL) in air or bubble (≈4 µL) in water were investigated by a contact‐angle system (JC2000D, Powereach, China). Every value was obtained by measuring five different positions in a same sample surface. XRD patterns were collected on a D8 Advanced X‐ray Diffractometer (Bruker, Germany) using Cu Kα radiation. The chemical composition of the as‐synthesized mesh was characterized by a XPS (AXIS ULtrabid, Kratos, England), using mono Al Kα operated at 150 W. The processes of the rough mesh absorbing a water droplet in air, the F‐rough mesh absorbing a bubble in water, the rough mesh intercepting bubbles in water, and the F‐rough mesh allowing bubbles to pass through were captured by a high‐speed camera (CAMMC1362, Mikrotron, Germany) with the maximal frame rate of 2000 fps. The processes of removing bubbles from water pipe and collecting underwater bubbles were captured by a camera (D7100, Nikon, Japan).

## Conflict of Interest

The authors declare no conflict of interest.

## Supporting information

Supplementary

Supplementary

Supplementary

Supplementary

Supplementary

Supplementary

Supplementary

Supplementary

Supplementary

Supplementary
