# Supplementary material for: Underwater Superaerophobic and Superaerophilic Nanoneedles‐Structured Meshes for Water/Bubbles Separation: Removing or Collecting Gas Bubbles in Water
Source: Glob Chall. 2018 Apr 25;2(4):1700133. doi: 10.1002/gch2.201700133 (PMC6607170; doi:10.1002/gch2.201700133)
Supplement: Supplementary file 1 — Supplementary [file GCH2-2-1700133-s002.pdf]

Copyright WILEY-VCH Verlag GmbH & Co. KGaA, 69469 Weinheim, Germany, 2018.

Supporting Information

for *Global Challenges,* DOI: 10.1002/gch2.201700133

Underwater Superaerophobic and Superaerophilic Nanoneedles-Structured Meshes for Water/Bubbles Separation: Removing or Collecting Gas Bubbles in Water

Jiale Yong, Feng Chen, Wentao Li, Jinglan Huo, Yao Fang, Qing Yang,* Hao Bian, and Xun Hou*

Copyright WILEY-VCH Verlag GmbH & Co. KGaA, 69469 Weinheim, Germany, 2013.

Supporting Information

Underwater Superaerophobic and Superaerophilic Nanoneedles-Structured Meshes for Water/Bubbles Separation: Removing or Collecting Gas Bubbles in Water

Jiale Yong, Feng Chen, Wentao Li, Jinglan Huo, Yao Fang, Qing Yang*, Hao Bian, and Xun Hou*

(Including Figures S1-S3 and Movies S1~S9 in the Supporting Information)

Movie S1. Water droplet spreading out on a rough mesh in air.

Movie S2. Immersing a rough mesh and an F-rough mesh into water.

Movie S3. Bubble rolling on a rough mesh in water.

Movie S4. Water droplet rolling on an F-rough mesh in air.

Movie S5. Bubble being absorbed by an F-rough mesh in water.

Movie S6. Bubbles being intercepted by the underwater rough mesh.

Movie S7. Bubbles passing through the underwater F-rough mesh.

Movie S8. Process of removing bubbles in a water pipe.

Movie S9. Process of collecting bubbles by the designed device.

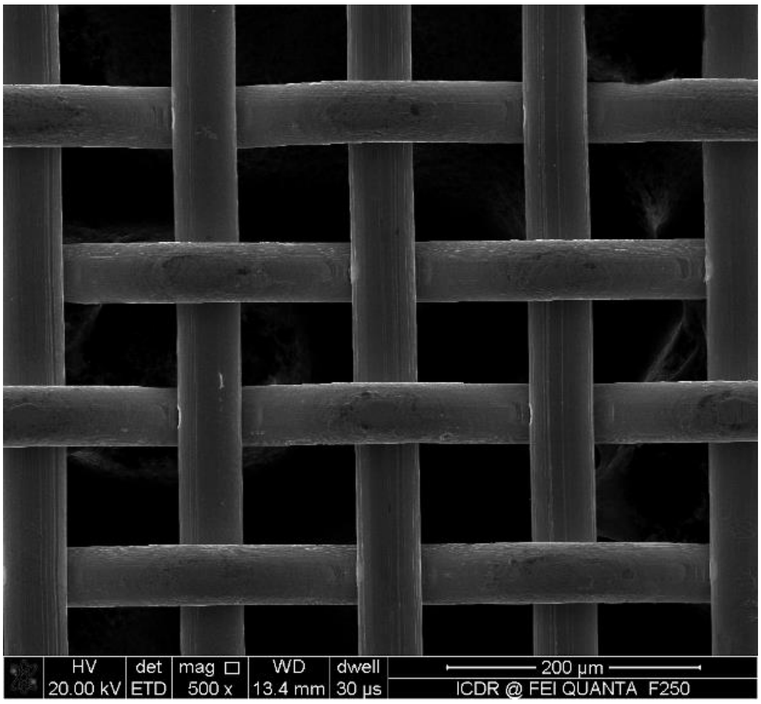

Figure S1. SEM image of the pure copper mesh.

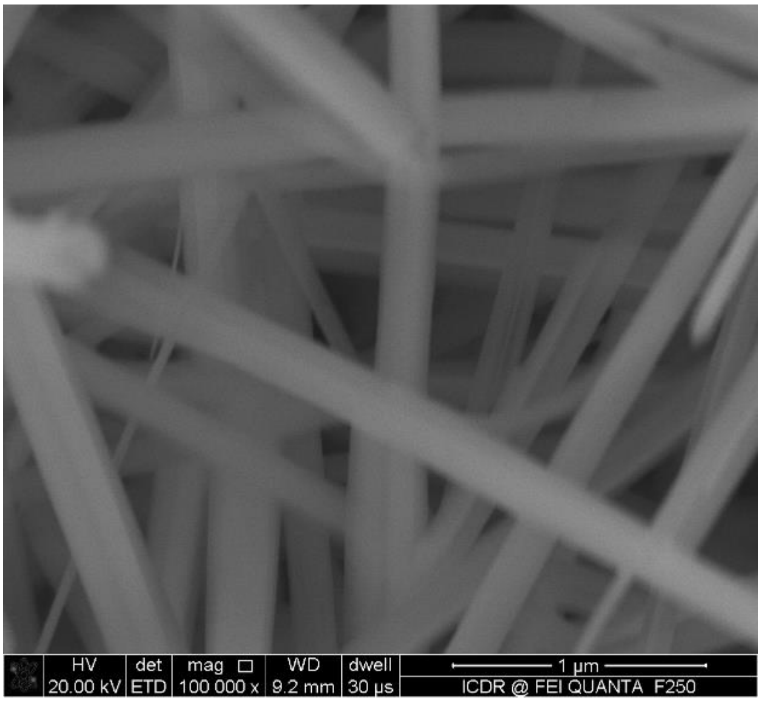

Figure S2. High-magnification SEM image of the nanoneedles structure.

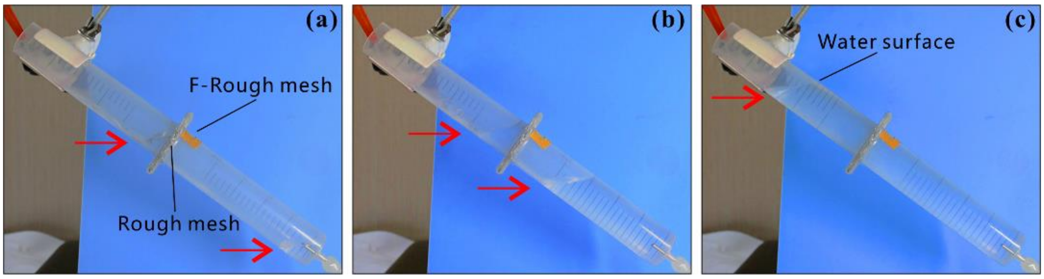

Figure S3. Pouring water into the artificial bubbles-removing water pipe. The red arrows show the location of the water surface.
